# Oridonin Attenuates Cisplatin-Induced Ovarian Injury by Modulating Oxidative Stress, Inflammation, and TGF-β1/Smad3-Mediated Fibrosis in Rats

**DOI:** 10.3390/medicina62071231

**Published:** 2026-06-25

**Authors:** Gulseren Dinc, Bakiye Akbas, Ahmet Akbas, Hatice Aygun, Oytun Erbas

**Affiliations:** 1 Department of Obstetrics and Gynecology, Faculty of Medicine, Karadeniz Technical University, Trabzon 61080, Turkey; bakiyeakbas@ktu.edu.tr; 2 Department of General Surgey, Faculty of Medicine, Karadeniz Technical University, Trabzon 61080, Turkey; ahmetakbas@ktu.edu.tr; 3 Neuroscience Laboratory, Biruni Advanced Technology Application and Research Center, Faculty of Medicine, Biruni University, Istanbul 34015, Turkey; hatice_5aygun@hotmail.com (H.A.); oytunerbas2012@gmail.com (O.E.)

**Keywords:** oridonin, cisplatin, ovarian reserve, oxidative stress, fibrosis

## Abstract

*Background and Objectives*: The aim of this study is to evaluate the effects of oridonin on a cisplatin-induced ovarian injury rat model. *Materials and Methods*: Thirty female rats were divided into three groups. Group 1: control; group 2: cisplatin; group 3: cisplatin plus oridonin group. In groups 2 and 3, the rats were injected with 2.5 mg/kg (twice weekly) cisplatin intraperitoneally (i.p.) for 4 weeks. In Group 3, rats received oridonin (10 mg/kg/day, i.p.). At the end of the study, the ovaries were removed in all groups. Histopathologic analysis and follicle counting were performed. Plasma anti-Müllerian hormone (AMH), malondialdehyde (MDA), and tumor necrosis factor-alpha (TNF-α) levels were measured, while ovarian transforming growth factor-beta 1 (TGF-β1), SMAD family member 3 (SMAD3), and tissue inhibitor of metalloproteinases-1 (TIMP-1) levels were evaluated. *Results*: Oridonin alleviated cisplatin-induced histopathological changes in the ovarian tissue. The numbers of primordial, primary, secondary, and tertiary follicles were significantly decreased, while ovarian fibrosis was significantly increased in Group 2 compared with Group 1 (*p* < 0.05). Co-treatment with oridonin statistically significantly increased follicle counts at all developmental stages and markedly reduced ovarian fibrosis in group 2 compared with group 3. Compared with Group 1, AMH decreased, whereas MDA, TNF-α, TGF-β1, SMAD3, and TIMP-1 increased in Group 2 (*p* < 0.001); these alterations were markedly attenuated in Group 3. *Conclusions*: These findings suggest that oridonin may exert protective effects against cisplatin-induced ovarian injury.

## 1. Introduction

Chemotherapy-induced ovarian damage has become an important clinical concern [1,2]. Over the past two decades, the rate of spontaneous premature ovarian insufficiency has remained relatively stable, whereas treatment-related cases have increased [3,4]. Each year, millions of women are diagnosed with cancer worldwide, and a substantial proportion are of reproductive age [5,6]. In these patients, exposure to chemotherapy and radiotherapy is strongly associated with ovarian failure and a high risk of subsequent fertility loss [7,8,9].

Cisplatin is a widely used platinum-based chemotherapeutic agent [10,11]. A limited number of studies have shown that cisplatin reduces ovarian follicle reserve, thereby compromising reproductive capacity [12,13,14].

Today, natural compounds have attracted increasing interest in the prevention and treatment of various diseases due to their low toxicity and wide range of biological effects. In particular, many studies have shown that plant-derived compounds may exert protective effects through their antioxidant and anti-inflammatory properties [12,15,16]. Oridonin is a natural compound isolated from the medicinal plant Isodon rubescens. Previous studies have shown that oridonin inhibits NF-κB activation, suppresses pro-inflammatory cytokine production, and modulates apoptosis-related pathways in various experimental models [17,18]. In addition, oridonin has been reported to reverse cisplatin resistance in ovarian cancer cells, suggesting a potential interaction with platinum-based chemotherapy [19]. Data on the ovarian effects of oridonin remain scarce and are largely limited to cancer-based models, with only a few studies focusing on granulosa cells [19,20,21,22,23]. Importantly, its potential role in cisplatin-induced ovarian injury has not yet been clarified.

Therefore, this study aimed to investigate whether oridonin could mitigate cisplatin-induced ovarian damage, with a particular focus on follicular reserve, oxidative stress, inflammation, and fibrosis.

## 2. Materials and Methods

### 2.1. Animals

A total of 30 adult female Wistar rats (200–210 g) were used. The animals were obtained from our institutional animal facility and housed under standard laboratory conditions (22 ± 2 °C, 12 h light/dark cycle) with free access to food and water. All procedures were approved by the local Animal Ethics Committee (Approval No: 53049415; Date: 21 April 2023). Cisplatin was obtained from Koçak Farma (Istanbul, Turkey) and freshly prepared in saline. Oridonin (A diterpenoid compound, Sigma-Aldrich Company, St. Louis, MO, USA) was initially dissolved in DMSO and subsequently diluted with saline to obtain a final vehicle containing 5% DMSO and 95% saline.

### 2.2. Study Protocol

**Figure 1:**
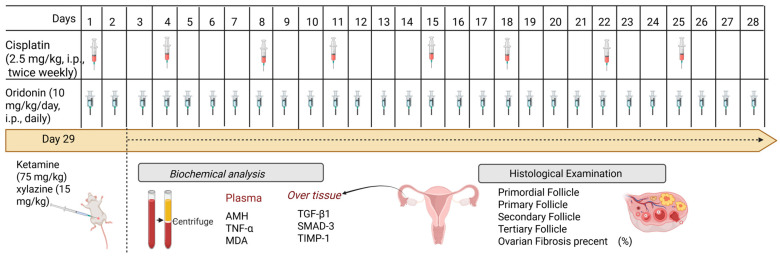
Experimental design of the study. Cisplatin (2.5 mg/kg, i.p.) was administered twice weekly, while oridonin (10 mg/kg/day, i.p.) was given daily for 28 days. On day 29, blood and ovarian tissues were collected under ketamine/xylazine anesthesia. Plasma AMH, TNF-α, and MDA levels were analyzed, and ovarian tissues were evaluated for TGF-β1, SMAD3, TIMP-1, follicle counts, and fibrosis.

A total of 30 rats were divided into three groups (n = 10 each). Cisplatin (2.5 mg/kg, i.p.) was administered twice weekly for four weeks (cumulative dose: 20 mg/kg) in Groups 2 and 3 (Figure 1).

Group 1 (Control): Rats received vehicle solution (5% DMSO/95% saline, 1 mL/kg/day, i.p.).

Group 2 (CP): Rats received cisplatin and vehicle solution (5% DMSO/95% saline, 1 mL/kg/day, i.p.).

Group 3 (CP + ORD): Rats received cisplatin and oridonin (10 mg/kg/day, i.p.) dissolved in 5% DMSO/95% saline for four weeks.

At the end of the study, animals were anesthetized with ketamine (75 mg/kg) and xylazine (15 mg/kg) and sacrificed. Blood samples were collected, and ovarian tissues were removed for histological and biochemical analyses.

### 2.3. Histological Examination

Ovarian tissues were fixed in formalin and embedded in paraffin. Sections (4 μm) were cut and stained with hematoxylin and eosin. The slides were examined under a light microscope.

Images were taken using an Olympus BX51 microscope (Olympus Corporation, Tokyo, Japan) equipped with a digital camera (Image-Pro Express, version 1.4.5; Media Cybernetics, Rockville, MD, USA). For each specimen, ten randomly selected fields were evaluated at ×20 magnification. All assessments were conducted by an observer blinded to the experimental groups.

Follicular classification was performed based on established morphological criteria:

• Primordial follicle: An oocyte surrounded by a single layer of flattened follicular epithelial cells
• Primary follicle: An oocyte encircled by a single layer of cuboidal granulosa cells
• Secondary follicle: A growing oocyte with two or more layers of granulosa cells, accompanied by zona pellucida formation and theca folliculi development
• Tertiary follicle: Characterized by the presence of a fluid-filled antral cavity, well-defined stratum granulosum, and cumulus oophorus

In addition, stromal fibrosis in ovarian tissue was quantified and expressed as a percentage.

### 2.4. Measurement of Plasma AMH and TNF-α Levels

Blood samples collected from the animals were centrifuged at 3000 rpm for 10 min at room temperature. Plasma samples were stored at −20 °C until analysis. AMH and TNF-α levels were measured by ELISA kits (Biosciences, Seattle, WA, USA). Samples were run in duplicate.

Plasma MDA levels were determined by the TBARS method. Briefly, samples were mixed with TCA and TBARS reagent, incubated at 100 °C, centrifuged, and absorbance was measured at 535 nm. Results were expressed as nmol/mL (Figure 1).

### 2.5. Ovarian Tissue Biochemical Analysis

After sacrifice, ovarian tissues were removed and stored at −20 °C until analysis. Samples were homogenized in phosphate-buffered saline (pH 7.4) and centrifuged (5000× *g*, 15 min). The supernatants were used for analysis. Total protein levels were measured by the Bradford method. TGF-β1, SMAD3, and TIMP-1 levels were determined by ELISA. Absorbance was read using a microplate reader (Multiskan GO, Thermo Fisher Scientific, Waltham, MA, USA).

### 2.6. Statistical Analysis

Statistical analyses were performed using SPSS version 23.0 (IBM Corp., Armonk, NY, USA). Graphs were generated using GraphPad Prism version 10.0 (GraphPad Software, San Diego, CA, USA). Data distribution was assessed using the Shapiro–Wilk test, and homogeneity of variances was evaluated using Levene’s test. Differences among groups were analyzed using one-way analysis of variance (ANOVA) followed by Tukey’s multiple-comparison post hoc test. Data are presented as mean ± standard error of the mean (SEM). A *p* value < 0.05 was considered statistically significant.

## 3. Results

### 3.1. Histopathological Evaluation

#### 3.1.1. Primordial Follicle

**Figure 2:**
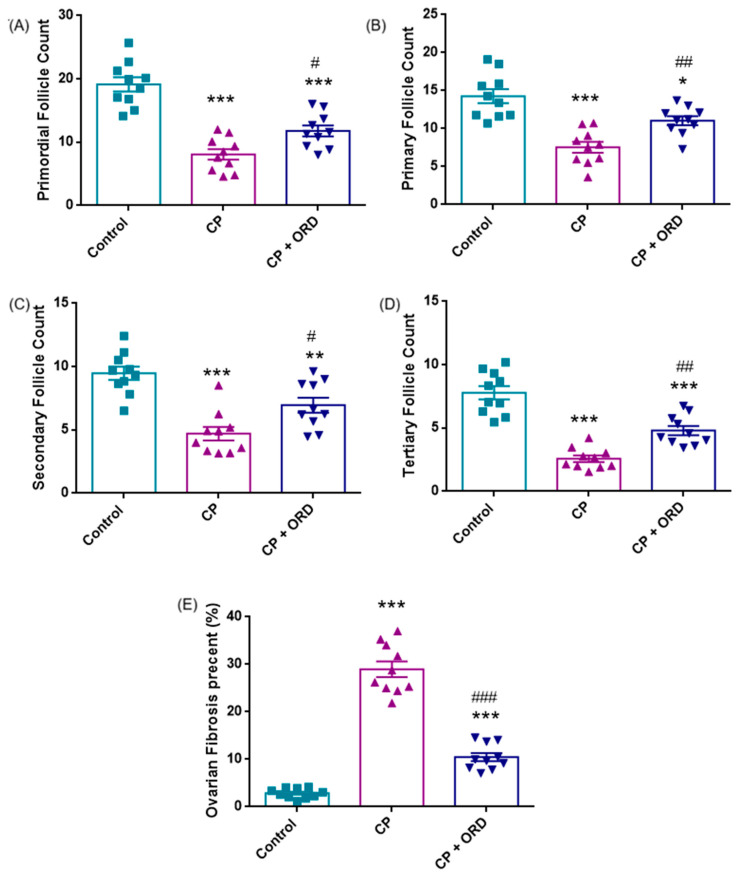
Effects of cisplatin and oridonin on ovarian histopathological parameters. (**A**) Primordial follicle count, (**B**) primary follicle count, (**C**) secondary follicle count, (**D**) tertiary follicle count, and (**E**) ovarian fibrosis percentage. Data are presented as mean ± SEM (n = 10). Statistical analysis was performed using one-way ANOVA followed by Tukey’s multiple-comparison test. *** *p* < 0.001, ** *p* < 0.01, * *p* < 0.05 vs. control group; # *p* < 0.05, ## *p* < 0.01, ### *p* < 0.001 vs. cisplatin group. CP: cisplatin; ORD: oridonin.

**Figure 3:**
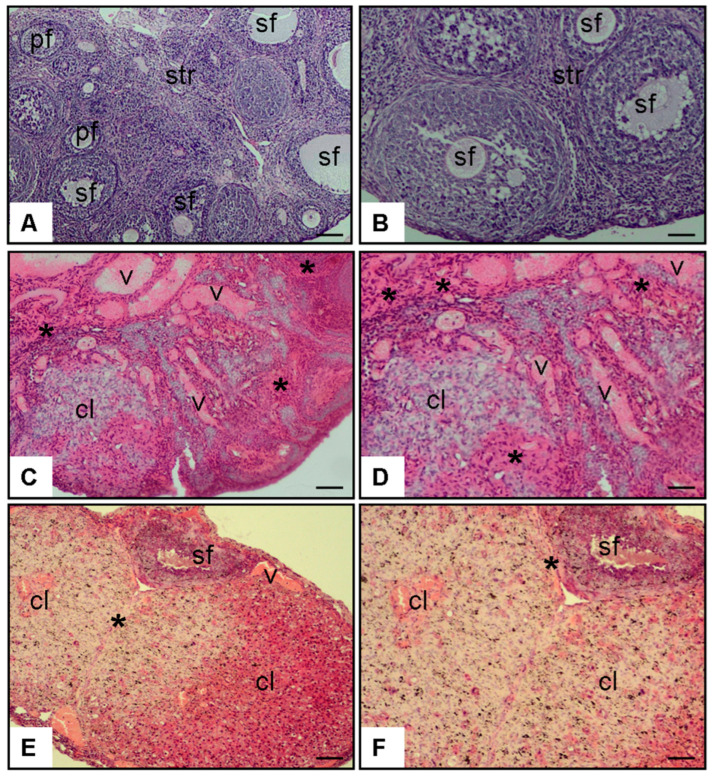
Representative hematoxylin and eosin (H&E)-stained ovarian sections (×10–×20). (**A**,**B**) Control group showing preserved ovarian architecture with normal stromal tissue (str), primary follicles (pf), secondary follicles (sf), vascular structures (v), and corpus luteum (cl). (**C**,**D**) Cisplatin-treated group showing marked stromal fibrosis (asterisks), tissue disorganization, follicular depletion, and disruption of normal ovarian architecture. (**E**,**F**) Cisplatin plus oridonin-treated group showing reduced stromal fibrosis (asterisks) and partial restoration of ovarian morphology. Asterisks indicate fibrotic areas. Scale bars = 100 μm.

As shown in Figure 2A and Figure 3A, primordial follicle counts differed significantly among the experimental groups (one-way ANOVA, F(2,27) = 35.00, *p* < 0.0001). The primordial follicle count was significantly lower in the cisplatin-treated group (Group 2; 8.08 ± 0.84) than in the control group (Group 1; 19.14 ± 1.12; *p* < 0.0001). Primordial follicle counts were significantly higher in the cisplatin + oridonin group (Group 3; 11.79 ± 0.86) than in the cisplatin group (Group 2; *p* = 0.0270), although values remained significantly lower than those of the control group (*p* < 0.0001).

#### 3.1.2. Primary Follicle

As shown in Figure 2B, primary follicle counts differed significantly among the experimental groups (one-way ANOVA, F(2,27) = 19.58, *p* < 0.0001). The primary follicle count was significantly lower in the cisplatin-treated group (Group 2; 7.54 ± 0.72) than in the control group (Group 1; 14.27 ± 0.93, *p* < 0.0001). Primary follicle counts were significantly higher in the cisplatin + oridonin group (Group 3; 11.04 ± 0.59) than in the cisplatin group (Group 2; *p* = 0.0084), although values remained significantly lower than those of the control group (*p* = 0.0152).

#### 3.1.3. Secondary Follicle

As shown in Figure 2C, secondary follicle counts differed significantly among the experimental groups (one-way ANOVA, F(2,27) = 18.71, *p* < 0.0001). The secondary follicle count was significantly lower in the cisplatin-treated group (Group 2; 4.70 ± 0.56) than in the control group (Group 1; 9.47 ± 0.53, *p* < 0.0001). Secondary follicle counts were significantly higher in the cisplatin + oridonin group (Group 3; 6.96 ± 0.60) than in the cisplatin group (Group 2; *p* = 0.0198), although values remained significantly lower than those of the control group (*p* = 0.0090).

#### 3.1.4. Tertiary Follicle

As shown in Figure 2D, tertiary follicle counts differed significantly among the experimental groups (one-way ANOVA, F(2,27) = 41.69, *p* < 0.0001). The tertiary follicle count was significantly lower in the cisplatin-treated group (Group 2; 2.57 ± 0.26) than in the control group (Group 1; 7.79 ± 0.53, *p* < 0.0001). Tertiary follicle counts were significantly higher in the cisplatin + oridonin group (Group 3; 4.80 ± 0.37) than in the cisplatin group (Group 2; *p* = 0.0017), although values remained significantly lower than those of the control group (*p* < 0.0001).

#### 3.1.5. Ovarian Fibrosis

As shown in Figure 2E, ovarian fibrosis percentages differed significantly among the experimental groups (one-way ANOVA, F(2,27) = 151.0, *p* < 0.0001). The ovarian fibrosis percentage was significantly higher in the cisplatin-treated group (Group 2; 28.97 ± 1.65%, Figure 3C,D) than in the control group (Group 1; 2.86 ± 0.33%, *p* < 0.0001, Figure 3A,B). Ovarian fibrosis percentages were significantly lower in the cisplatin + oridonin group (Group 3; 10.47 ± 0.86%, Figure 3E,F) than in the cisplatin group (Group 2; *p* < 0.0001), although fibrosis levels remained significantly higher than those of the control group (*p* = 0.0001).

### 3.2. Biochemical Findings

#### 3.2.1. Plasma AMH

**Figure 4:**
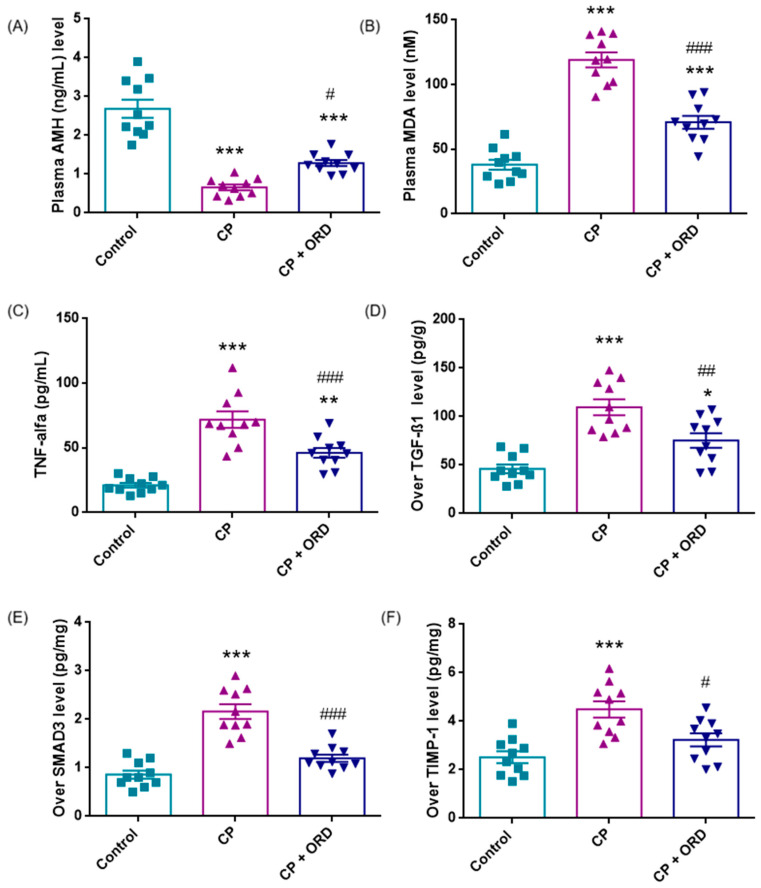
Effects of cisplatin and oridonin on plasma and ovarian biochemical parameters. (**A**) Plasma AMH, (**B**) plasma MDA, (**C**) TNF-α, (**D**) ovarian TGF-β1, (**E**) ovarian SMAD3, and (**F**) ovarian TIMP-1 levels. Data are presented as mean ± SEM (n = 10). Statistical analysis was performed using one-way ANOVA followed by Tukey’s multiple-comparison test. *** *p* < 0.001, ** *p* < 0.01, * *p* < 0.05 vs. control group; # *p* < 0.05, ## *p* < 0.01, ### *p* < 0.001 vs. cisplatin group. CP: cisplatin; ORD: oridonin.

As shown in Figure 4A, plasma AMH levels differed significantly among the experimental groups (one-way ANOVA, F(2,27) = 48.76, *p* < 0.0001). Plasma AMH levels were significantly lower in the cisplatin-treated group (Group 2; 0.66 ± 0.07 ng/mL) than in the control group (Group 1; 2.69 ± 0.23 ng/mL, *p* < 0.0001), representing a 75.6% decrease. Plasma AMH levels were significantly higher in the cisplatin + oridonin group (Group 3; 1.29 ± 0.08 ng/mL) than in the cisplatin group (Group 2; *p* = 0.0178), corresponding to a 95.9% increase, although values remained significantly lower than those of the control group (*p* < 0.0001).

#### 3.2.2. Plasma MDA

As shown in Figure 4B, plasma MDA levels differed significantly among the experimental groups (one-way ANOVA, F(2,27) = 68.64, *p* < 0.0001). Plasma MDA levels were significantly higher in the cisplatin-treated group (Group 2; 119.10 ± 5.81 nM) than in the control group (Group 1; 38.07 ± 3.87 nM, *p* < 0.0001), representing a 212.9% increase (3.1-fold increase). Plasma MDA levels were significantly lower in the cisplatin + oridonin group (Group 3; 70.88 ± 4.89 nM) than in the cisplatin group (Group 2; *p* < 0.0001), corresponding to a 40.5% decrease, although values remained significantly higher than those of the control group (*p* = 0.0002).

#### 3.2.3. Plasma TNF-α

As shown in Figure 4C, plasma TNF-α levels differed significantly among the experimental groups (one-way ANOVA, F(2,27) = 33.36, *p* < 0.0001). Plasma TNF-α levels were significantly higher in the cisplatin-treated group (Group 2; 71.86 ± 6.36 pg/mL) than in the control group (Group 1; 21.11 ± 1.78 pg/mL, *p* < 0.0001), representing a 240.4% increase (3.4-fold increase). Plasma TNF-α levels were significantly lower in the cisplatin + oridonin group (Group 3; 46.21 ± 3.79 pg/mL) than in the cisplatin group (Group 2; *p* = 0.0009), corresponding to a 35.7% decrease, although values remained significantly higher than those of the control group (*p* = 0.0011).

#### 3.2.4. Ovarian TGF-β1

As shown in Figure 4D, ovarian TGF-β1 levels differed significantly among the experimental groups (one-way ANOVA, F(2,27) = 20.74, *p* < 0.0001). Ovarian TGF-β1 levels were significantly higher in the cisplatin-treated group (Group 2; 109.50 ± 8.29 pg/g) than in the control group (Group 1; 46.07 ± 4.52 pg/g, *p* < 0.0001), representing a 137.7% increase. Ovarian TGF-β1 levels were significantly lower in the cisplatin + oridonin group (Group 3; 75.31 ± 7.53 pg/g) than in the cisplatin group (Group 2; *p* = 0.0049), corresponding to a 31.2% decrease, although values remained significantly higher than those of the control group (*p* = 0.0167).

#### 3.2.5. Ovarian SMAD3

As shown in Figure 4E, ovarian SMAD3 levels differed significantly among the experimental groups (one-way ANOVA, F(2,27) = 38.71, *p* < 0.0001). Ovarian SMAD3 levels were significantly higher in the cisplatin-treated group (Group 2; 2.16 ± 0.15 pg/mg) than in the control group (Group 1; 0.86 ± 0.08 pg/mg, *p* < 0.0001), representing a 151.2% increase (2.5-fold increase). Ovarian SMAD3 levels were significantly lower in the cisplatin + oridonin group (Group 3; 1.20 ± 0.08 pg/mg) than in the cisplatin group (Group 2; *p* < 0.0001), corresponding to a 44.6% decrease. No significant difference was observed between the cisplatin + oridonin group and the control group (*p* = 0.0910).

#### 3.2.6. Ovarian TIMP-1

As shown in Figure 4F, ovarian TIMP-1 levels differed significantly among the experimental groups (one-way ANOVA, F(2,27) = 12.19, *p* = 0.0002). Ovarian TIMP-1 levels were significantly higher in the cisplatin-treated group (Group 2; 4.49 ± 0.34 pg/mg) than in the control group (Group 1; 2.52 ± 0.24 pg/mg, *p* = 0.0001), representing a 78.2% increase. Ovarian TIMP-1 levels were significantly lower in the cisplatin + oridonin group (Group 3; 3.23 ± 0.27 pg/mg) than in the cisplatin group (Group 2; *p* = 0.0118), corresponding to a 28.0% decrease. No significant difference was observed between the cisplatin + oridonin group and the control group (*p* = 0.2002).

## 4. Discussion

This study revealed that the administration of cisplatin in rats caused ovarian damage by inflammation, stimulating oxidative stress, and fibrotic activity. Histological and biochemical analyses showed that the oridonin co-treatments attenuated cisplatin-induced ovarian damage. These results indicated that oridonin has the potential to abolish cisplatin-induced ovo-toxicity.

Chemotherapy is known to affect ovarian function, and the extent of damage depends on several factors such as age, drug type, dose, and treatment protocol [24]. Cisplatin, a commonly used chemotherapeutic agent, has been reported in both clinical and experimental studies to induce ovarian damage and follicle loss.

Histopathological analysis is considered the gold standard in the determination of ovarian reserve. Our histopathological results were similar to those reported in previous experimental models of chemotherapy-induced ovarian damage. In our study, follicle numbers were reduced at all stages in the cisplatin group, and ovarian fibrosis was increased. Similar findings have been reported in experimental studies, showing that cisplatin causes follicular loss and stromal damage [13,25]. In this study, the cisplatin + oridonin group exhibited higher follicle counts across developmental stages and higher AMH levels than the cisplatin group, suggesting partial recovery of ovarian function.

AMH is primarily produced by granulosa cells of preantral and small antral follicles and closely reflects the size of the growing follicle pool [26]. Therefore, the higher AMH levels observed in the cisplatin + oridonin group may be consistent with preservation of granulosa cell integrity and follicular function rather than a transient endocrine effect [12,27]. Similar findings have been reported with protective agents such as melatonin, supporting the interpretation that these changes reflect true tissue recovery [28,29]. Notably, to the best of our knowledge, no experimental or clinical studies have directly examined the effect of oridonin on AMH levels. Therefore, the increase in AMH observed in the present study may provide preliminary evidence of a previously unexplored link between oridonin and ovarian reserve. However, granulosa cell apoptosis or survival markers were not evaluated, and therefore the underlying mechanism could not be directly confirmed.

The antifibrotic findings represent one of the key strengths of this study. Chemotherapy-associated ovarian injury is increasingly recognized as a redox-driven process in which excessive ROS damages granulosa cells, promotes lipid peroxidation and ferroptotic/apoptotic injury, and is accompanied by stromal remodeling and fibrosis [13,25]. In cisplatin-based models, oxidative damage and ferroptosis have been closely linked to ovarian fibrosis, and interventions targeting oxidative stress or TGF-β/Smad signaling have been shown to preserve follicle pools and ovarian structure [9,25,30]. These findings support our observations and highlight the central role of oxidative stress-driven fibrotic signaling in cisplatin-induced ovarian injury. Nevertheless, ferroptosis-related markers such as GPX4 and ACSL4 were not evaluated in the present study, and therefore the potential involvement of ferroptosis could not be directly confirmed. In line with this, oridonin has been reported to enhance antioxidant responses in various experimental models [15,16,17,18], and similar protective effects have been described in cisplatin-induced organ injury, supporting a broader role for oridonin against platinum-related toxicity [31].

In our study, TNF-α levels were higher in the cisplatin group, suggesting increased inflammatory activity. TNF-α seems to play an upstream role in this process. Previous study showed that TNF-α can increase TGF-β1 expression through ERK/AP-1 signaling and may enhance tissue responsiveness to TGF-β, leading to stronger Smad signaling [32]. Increased TGF-β1/Smad3 activity has been linked to stromal fibrosis and impaired ovarian function, while its inhibition has been associated with structural improvement [30,33]. Shi et al. [13] reported that TGF-β1 promotes fibrosis in ovarian tissue via Smad2/3 signaling. In addition, previous studies have shown that TGF-β1 regulates TIMP-1 expression through the Smad pathway. Increased TGF-β1 activity has been associated with higher TIMP-1 levels, which may reduce extracellular matrix degradation and contribute to fibrosis.

In the present study, oridonin reduced ovarian fibrosis and was associated with decreased TGF-β1, SMAD3, and TIMP-1 levels, suggesting suppression of profibrotic signaling rather than a purely morphological effect [30,31]. These findings align with the broader pharmacological profile of oridonin, which has been shown to reduce oxidative stress, suppress inflammation, and inhibit TGF-β/Smad-mediated fibrosis in multiple organ systems [17,31,34,35]. Taken together, the parallel reduction in circulating MDA and TNF-α and ovarian TGF-β1/SMAD3/TIMP-1 levels after oridonin treatment is biologically coherent and suggests interruption of a TNF-α–TGF-β1/Smad3–TIMP-1 axis that would otherwise sustain fibrosis and follicle loss. Although these findings support a potential TNF-α–TGF-β1/Smad3–TIMP-1 signaling axis, the present data are correlative and do not establish causality. Further studies using pathway-specific inhibitors or genetic approaches are needed to confirm these mechanistic relationships.

A limitation of the present study is the absence of an oridonin-only group. Therefore, the observed findings should be interpreted as protective effects of oridonin in the context of cisplatin-induced ovarian injury rather than as evidence of its independent effects on ovarian physiology.

## 5. Conclusions

We conclude that oridonin may have a protective effect against cisplatin-induced ovarian damage. Follicle counts were higher, fibrosis was lower, and AMH levels were improved in the treatment group. These findings were consistent with changes in oxidative stress and inflammatory markers, suggesting a role in modulating both inflammation and tissue remodeling. However, further studies are needed to clarify the underlying mechanisms and to determine whether similar effects can be observed in humans. Future studies should evaluate fertility outcomes, pregnancy rates, dose–response relationships, and long-term safety profiles.

## Data Availability

All data are available upon request from the corresponding author.

## Abbreviations

The following abbreviations are used in this manuscript:

AMH	Antimüllerian hormone
TNF-α	TNF-α Tumor necrosis factor-α
MDA	Malondialdehyde
CP	Cisplatin
ORD	Oridonin
TGF-β1	Transforming growth factor--β1
SMAD3	Mothers against decapentaplegic homolog 3
TIMP-1	Tissue Inhibitor of Metalloproteinases-1
